# Actual survival after resection of primary colorectal cancer: results from a prospective multicenter study

**DOI:** 10.1186/s12957-021-02207-4

**Published:** 2021-04-05

**Authors:** Inge van den Berg, Robert R. J. Coebergh van den Braak, Jeroen L. A. van Vugt, Jan N. M. Ijzermans, Stefan Buettner

**Affiliations:** Department of Surgery, Erasmus MC - University Medical Center Rotterdam, Rotterdam, 3015 GD The Netherlands

## Abstract

**Background:**

Colorectal cancer is the third most common type of cancer in the world. We characterize a cohort of patients who survived up to 5 years without recurrence and identify factors predicting the probability of cure.

**Methods:**

We analyzed data of patients who underwent curative intent surgery for stage I–III CRC between 2007 and 2012 and who had had been included in a large multicenter study in the Netherlands. Cure was defined as 5-year survival without recurrence. Survival data were retrieved from a national registry.

**Results:**

Analysis of data of 754 patients revealed a cure rate of 65% (*n* = 490). Patients with stage I disease and T1- and N0-tumor had the highest probability of cure (94%, 95% and 90%, respectively). Those with a T4-tumor or N2-tumor had the lowest probability of cure (62% and 50%, respectively). A peak in the mortality rate for older patients early in follow-up suggests early excess mortality as an explanation. A similar trend was observed for stage III disease, poor tumor grade, postoperative complications, sarcopenia, and R1 resections. Patients with stage III disease, poor tumor grade, postoperative complications, sarcopenia, and R1 resections show a similar trend for decrease in CSS deaths over time.

**Conclusion:**

In the studied cohort, the probability of cure for patients with stage I–III CRC ranged from 50 to 95%. Even though most patients will be cured from CRC with standard therapy, standard therapy is insufficient for those with poor prognostic factors, such as high T- and N-stage and poor differentiation grade.

## Introduction

With an incidence of over 1.8 million new cases and almost 861,000 deaths in 2018 according to the World Health Organization, colorectal cancer (CRC) is the third most common cancer in the world [1]. Currently, the American Joint Committee on Cancer (AJCC) TNM classification is the most important determinant for treatment decisions and outcome. The standard treatment for stage I–III colon cancer is surgical resection of the primary tumor for patients, which is associated with a 5-year survival rate ranging from 92% in stage 1 to 53% in stage III [2]. Still, clinical outcomes of individual patients with resectable tumors vary. Besides tumor characteristics, patient factors such as obesity, diabetes mellitus, smoking, and nutritional status have been associated with survival, yet much of the disparity in prognosis remains unexplained [3–5].

Recurrence of CRC is chiefly a time-limited phenomenon, as 60–80% of recurrences becoming apparent within the first 2 years after resection and 95% within the first 4 years after resection [6]. The chances of recurrence remote after a 5-year recurrence-free period. Although recurrence is still possible after 5 years, the medical community considers many cancers “cured” when recurrence has not occurred within 5 years after diagnosis [7]. Owing to the considerable progress in the treatment in CRC during the past few decades, more and more patients remain free from recurrent disease after surgery [8, 9]. Even though the ideal intensity of follow-up is being debated [10, 11], the recurrence rate has been shown to reach a plateau phase 5 years after resection of the primary tumor. This is why follow-up programs in the Netherlands and many other countries have been limited to 5 years [10, 12–14]. In this multicenter study in a large Dutch colorectal cancer population, we sought to characterize the patients who survive up to 5 years without recurrence of disease and identify factors that affect probability of cure.

## Methods

### Study population

We analyzed data of patients with stage I–III colorectal cancer who had undergone curative intent surgery and had between 2007 and 2012 been enrolled in the MATCH-study, a prospective observational cohort study in patients undergoing curative resection for primary colorectal cancer in seven centers in the region of Rotterdam, the Netherlands [15]. The purpose of the MATCH study was to identify subtypes of colorectal cancer, related prognostic markers and outcome of treatment [16]. The MATCH study was approved by the Erasmus MC medical ethics review board (MEC-2007-088), and all patients provided written informed consent. All patients enrolled between 2007 and 2012 had the potential for 5 years of follow-up.

### Patient work-up and follow-up

#### Work-up

All patients underwent colonoscopy with a biopsy of any suspicious lesions. After tissue diagnosis was confirmed, laboratory studies were done with a goal of assessing patients’ organ function (liver, kidneys) in anticipation of diagnostic and therapeutic procedures and also to estimate tumor burden. Adequate imaging of the chest and abdomen was obtained for staging purposes. For colon cancer patients, this consisted of CT-abdomen and X-thorax and ultrasound of the liver when indicated. For rectal cancer patients, this consists of CT-thorax/abdomen and MRI rectum/pelvis.

Further work-up was driven by clinical setting, patient functional status and comorbidities and presenting symptoms. After adequate staging, adjuvant chemotherapy was offered for patients with high risk stage II and stage III colon cancer. At the time of this study, standard treatment consisted of 6 months CAPOX or FOLFOX. For rectal cancer patients, preoperative radiotherapy was offered for patients with T2–T4 tumors. For rectal cancer patients with positive CRM, or ≥ 4 positive lymph nodes, a combination of neoadjuvant radiotherapy and chemotherapy was offered.

#### Follow-up

CEA monitoring was performed 3-to-6 monthly in the first 3 years and 6-to-12 monthly hereafter and ultrasound of the liver or an abdominal CT every 6 months in the first 1–2 years and yearly hereafter. For rectal cancer patients, an additional x-thorax or CT-thorax could be considered, depending on the stage.

### Observed cure and follow-up status

Observed cure was defined as actual 5-year survival with no recurrence [6, 7, 13, 14]. At last follow-up, a patient was classified as having no evidence of disease (NED) if having survived without documented recurrence and as having died of disease (DOD) if the cause of death was listed as cancer in the national death registry. A patient classified as death of other cause (DOC) if a clearly attributable non-cancer reason for death was mentioned in the registry of Statistics Netherlands (*Centraal Bureau voor de Statistiek;* CBS). A patient was classified as dead of unknown cause (DUC) if no identifiable cause of death was found in the registry of Statistics Netherlands. A 5-year survivor with evidence of recurrent disease in the medical record was classified as alive with disease (AWD) [17].

### Predictors and outcome measures

Demographic variables included age and gender. Clinical variables included BMI, American Society of Anesthesiologists (ASA) score, International Union Against Cancer tumor node metastasis (TNM) classification of malignant tumors, tumor differentiation grade, tumor location, comorbidities, Charlson comorbidity index, and sarcopenia [18]. Treatment variables included the following: radicality, (neo)adjuvant therapy, postoperative complications classified according to Clavien-Dindo, and readmissions < 30 days.

To identify characteristics that may preclude long-term survival and cure, we compared the frequencies of these factors between specific survival cohorts defined as less than 1, 1 to 3, 3 to 5, and more than 5 years [17].

Cancer-specific survival (CSS) was calculated from the day of surgery to the day of death (from disease) or loss to follow-up, whichever came first. Date and cause of death were obtained from the national registry of Statistics Netherlands (*Centraal Bureau voor de Statistiek;* CBS). Patients who died of other causes than CRC were censored at the date of last follow-up. CSS was estimated using Kaplan-Meier methods and compared using log-rank.

### Statistical methods

The standard Cox proportional hazard model assumes proportional hazards, an assumption that can fail when survival curves have plateaus at the tails [19]. Hence, a semi-parametric proportional hazards mixture cure model was used to estimate the probability of cure and assess differences in outcome between cured patients and those who were not cured. In this model, the probability of being cured was modeled with logistic regression and the survival probability for patients who experienced the event of interest was estimated using a proportional hazards model [20–22]. All analyses were performed using the smcure package in R. v.3.3.2 (R foundation for Statistical Computing, Vienna, Austria) [22]. Two-sided *p* values < 0.05 were considered statistically significant.

## Results

### Patient characteristics and follow-up status

**Fig. 1:**
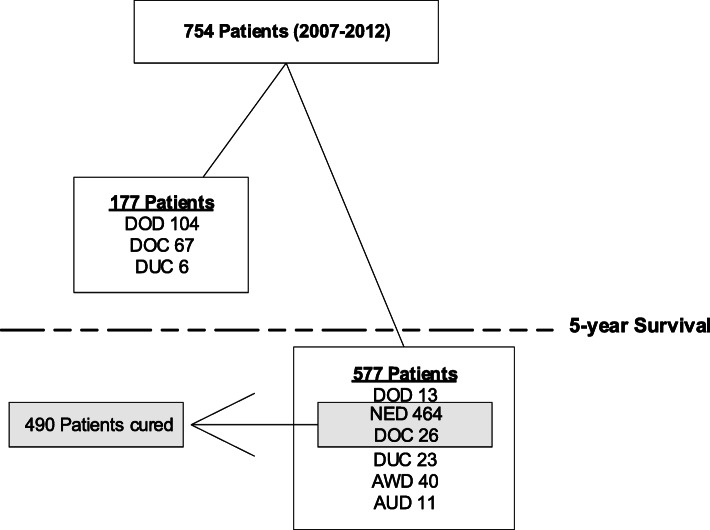
Current status and observed cure in study population

A total of 754 patients included in the MATCH study underwent surgical resection with curative intent in the period 2007 through 2012 (Fig. 1). At last follow-up, 117 patients (15.2%) could be classified as DOD, 40 (5.3%) as AWD, and 11 (1.5%) as AUD. In total, 93 patients (12.3%) could be classified as DOC and 29 (3.8%) as DUC. Data of the latter were excluded from CSS analyses. After 5 years of follow-up, 464 patients (61.5%) could be classified as NED and 26 (3.4%) died of a non-cancer related cause (DOC). These patients are considered cured from disease (NED + DOC> 5Y; *n* = 490) (Fig. 1).

### Cancer-specific survival through follow-up

Table 1 reports descriptive analyses on characteristics of patients over time. Patients were grouped by survival into less than 1 year survival, 1–3 years, 3–5 years, more than 5-years survival, and an additional category cured. The first three groups include only DOD patients; the non-cured group > 5 years consists of AWD, DOD, and AUC patients. The cured patients included 52.2% men, and 42.9% were aged ≥ 70. The latter relatively more often had died of CRC in the early years following surgery than had patients < 70 years, as can be seen in Table 1 from the decreasing proportion of older aged patients dying of CRC over time and increasing proportions of younger patients dying over time. Patients with stage III colon cancer, poor tumor grade, postoperative complications, sarcopenia, and or an incomplete resection margin show a similar trend for decrease in CSS deaths over time (Table 1).

**Table 1:** Comparison of prognostic factors among survival cohorts

	0–1	%	1–3	%	3–5	%	> 5^**a**^	%	Cured	%	Missing %
**Gender**											0
Male	18	(64.3)	31	(63.3)	13	(48.1)	34	(53.1)	256	(52.2)	
Female	10	(35.7)	18	(36.7)	14	(51.9)	30	(46.9)	234	(47.8)	
**Age**											0
< 70	5	(17.9)	17	(34.7)	11	(40.7)	31	(48.4)	280	(57.1)	
≥ 70	23	(82.1)	32	(65.3)	16	(59.3)	33	(51.6)	210	(42.9	
**BMI**											1.1
1 (< 18.5)	1	(3.7)	1	(2.0)	1	(3.7)	0	(0.0)	15	(3.1)	
2 (18.5–24.9)	10	(37.0)	22	(44.9)	10	(37.)	20	(32.3)	181	(37.3)	
3 (≥ 25.0)	16	(59.3)	26	(53.1)	16	(59.3)	42	(67.7)	289	(59.6)	
**Sarcopenia**											18.8
No	7	(31.8)	15	(41.7)	10	(47.6)	23	(50.0)	211	(51.8)	
Yes	15	(68.2)	21	(58.3)	11	(52.4)	23	(50.0)	196	(48.2)	
**Low muscle density**											19.6
No	5	(23.8)	11	(40.6)	6	(28.6)	13	(29.5)	159	(39.4)	
Yes	17	(76.2)	25	(69.4)	15	(71.4)	31	(70.5)	245	(60.6)	
**Sarcopenia + obese**											0
No	26	(92.9)	44	(89.8)	26	(96.3)	63	(98.4)	465	(94.9)	
Yes	2	(7.1)	5	(10.2)	1	(3.7)	1	(1.6)	25	(5.1)	
**Diabetes mellitus**											0.1
No	22	(78.6)	41	(83.7)	20	(76.9)	48	(75.0)	402	(82.0)	
Yes	6	(21.4)	8	(16.3)	6	(23.1)	16	(25.0)	88	(18.0)	
**Congestive heart failure**											0
No	25	(89.3)	45	(91.8)	24	(88.9)	63	(98.3)	466	(95.1)	
Yes	3	(10.7)	4	(8.2)	3	(11.1)	1	(1.6)	24	(4.9)	
**COPD**											0
No	27	(96.4)	44	(89.8)	23	(85.2)	62	(96.9)	454	(92.7)	
Yes	1	(3.6)	5	(10.2)	4	(14.8)	2	(3.1)	26	(7.3)	
**Charlson comorbidity index**											0.4
0	11	(39.3)	23	(46.9)	10	(38.5)	39	(60.9)	266	(54.4)	
1+	17	(60.7)	26	(53.1)	16	(61.5)	25	(39.1)	223	(45.6)	
**ASA score**											
I–II	22	(78.6)	45	(91.8)	20	(74.1)	54	(85.7)	409	(84.2)	
III–IV	6	(21.4)	4	(8.2)	7	(25.9)	9	(14.3)	77	(15.8)	
**CEA**											8.6
< 7 μg/L	21	(90.8)	28	(59.6)	11	(45.8)	36	(62.1)	360	(79.5)	
≥ 7 μg/L	5	(19.2)	19	(40.4)	13	(54.2)	22	(37.9)	93	(20.5)	
**Tumor location**											
Colon	20	(71.4)	31	(63.3)	14	(51.9)	41	(64.1)	371	(75.7)	0
Rectum	8	(28.6)	18	(36.7)	13	(48.1)	23	(35.9)	119	(24.3)	
**T-stage**											0
1	2	(7.1)	0		1	(3.7)	2	(3.1)	34	(6.9)	
2	7	(25)	5	(10.2)	7	(25.9)	22	(34.3)	171	(34.9)	
3	16	(57.1)	38	(77.6)	17	(63)	36	(56.2)	270	(55.1)	
4	3	(10.7)	6	(12.2)	2	(7.4)	4	(6.2)	15	(3.1)	
**N-stage**											0
0	8	(28.6)	13	(26.5)	6	(22.2)	36	(56.2)	303	(61.8)	
1	5	(17.9)	10	(20.4)	7	(25.9)	11	(17.2)	103	(21)	
2	12	(42.9)	21	(42.9)	7	(25.9)	7	(25.9)	33	(6.7)	
**X**	**3**	(10.7)	5	(10.4)	7	(25.9)	10	(15.6)	51	(10.4)	
**Tumor stage**											0
I	7	(25.0)	4	(8.2)	5	(18.5)	22	(34.4)	164	(33.5)	
II	4	(14.3)	14	(28.6)	8	(29.6)	24	(37.5)	190	(38.8)	
III	17	(60.7)	31	(63.3)	14	(51.9)	18	(28.1)	136	(27.8)	
**Tumor grade**											1.5
Good	1	(3.7)	5	(10.4)	3	(11.1)	10	(15.9)	66	(13.7)	
Moderate	16	(59.3)	36	(75.0)	21	(77.8)	47	(74.6)	377	(78.1)	
Poor	8	(29.6)	7	(14.6)	3	(11.1)	5	(7.9)	33	(6.8)	
Unknown/other	2	(7.4)	0		0		1	(1.6)	7	(1.4)	
**Radicality**											0.3
R0	26	(92.6)	48	(98.0)	27	(100.0)	63	(98.4)	477	(97.5)	
R1	2	(7.1)	1	(2.0)	0		1	(1.6)	12	(2.5)	
**Postoperative complications**											0
No	6	(21.4)	24	(49.0)	8	(29.6)	38	(59.4)	298	(60.8)	
Yes	22	(78.6)	25	(51.)	19	(70.4)	26	(40.6)	193	(39.2)	
**Readmission < 30 days**											0.1
No	24	(88.9)	47	(95.9)	23	(85.2)	55	(85.9)	438	(89.4)	
Yes	3	(11.1)	2	(4.1)	4	(14.8)	9	(14.1)	52	(10.6)	
**Neoadjuvant chemotherapy**											0
No	20	(71.4)	33	(59.3)	16	(59.3)	43	(67.2)	383	(78.3)	
Yes	8	(28.6)	16	(32.7)	11	(40.7)	21	(32.8)	106	(21.7)	
**Adjuvant chemotherapy**											0
No	28	(100)	37	(77.1)	22	(81.5)	55	(85.9)	382	(78.6)	
Yes	0		11	(22.9)	5	(18.5)	9	(14.1)	104	(21.4)	

^a^Non-cured group > 5 years consists of AWD, DOD, and AUC patients

**Fig. 2:**
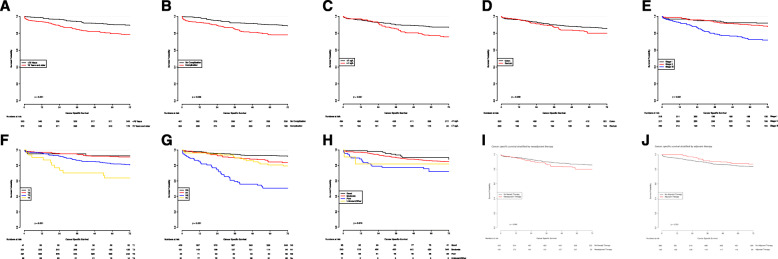
Kaplan-Meier curves on cancer-specific survival stratified by prognostic clinical factors. **a** Cancer-specific survival stratified by age. **b** Cancer-specific survival stratified by postoperative complications. **c** Cancer-specific survival stratified by preoperative CEA level. **d** Cancer-specific survival stratified by tumor location. **e** Cancer-specific survival stratified by tumor stage. **f** Cancer-specific survival stratified by T-stage. **g** Cancer-specific survival stratified by N-stage. **h** Cancer-specific survival stratified by tumor differentiation grade. **i** Cancer-specific survival stratified by neoadjuvant therapy. **j** Cancer-specific survival stratified by adjuvant therapy

Kaplan-Meier survival analyses were performed to determine which characteristics were associated with CSS. It appeared that age > 70 (*p* < 0.001), preoperative CEA level ≥ 7 μg/L (*p* = 0.001), high T-stage (*p* < 0.001), high N-stage (*p* < 0.001), high tumor stage (*p* < 0.001), poor tumor differentiation (*p* = 0.010), rectal cancer (*p* = 0.039), and occurrence of postoperative complications (*p* = 0.039) were all significantly associated with shorter CSS (Table 1; Fig. 2). It should be noted that from the patients with an N0 tumor, a total of 85 patients had < 10 lymph nodes dissected which could have led to wrong nodal staging.

### Observed cure and predicted cure

**Fig. 3:**
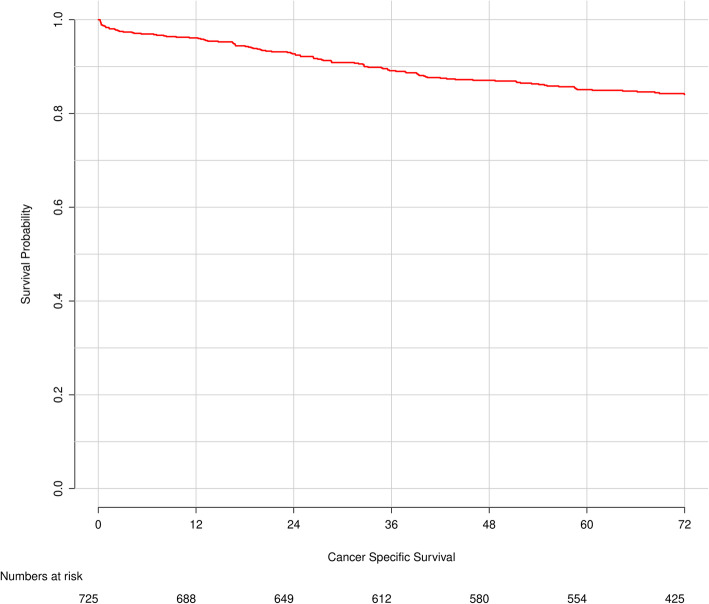
Kaplan-Meier curve for cancer-specific survival for all patients undergoing resection for stage I–III colorectal cancer

The potential survival cohort in Table 2 includes the 621 patients categorized as NED, DOD, or AWD. Overall, 577 patients survived 5 years. Most of them had been classified as NED (*n* = 464, 80.4%) and a small minority (*n* = 26, 4.5%) as DOC. These two groups are considered cured from disease; thus, the observed cure rate was 65% (490/754). At 5 years follow-up, 40 patients were classified as AWD and 13 as DOD. Data on recurrence were missing for 11 patients alive at 5 years (AUD). The CSS for the whole study cohort is visualized by a Kaplan-Meier curve (Fig. 3).

**Table 2:** Characteristics of patients with potential cure and probability of cure estimated from the semiparametric mixture cure model

	Total NED, DOD, AWD (***N*** = 621)	Observed 5-year survivors	% observed	Predicted cure
**Gender**				
Male	335	256	76.4	82
Female	286	234	81.8	83.7
**Age**				
< 70	340	280	82.4	88.5
≥ 70	281	210	74.7	80.1
**BMI**				
1 (< 18.5)	17	15	88.2	75.6
2 (18.5–24.9)	228	181	79.4	85.5
3 (≥ 25.0)	368	289	87.5	84
**Sarcopenia**				
No	254	211	83.1	86
Yes	246	196	79.7	82.8
**Low muscle density**				
No	190	159	83.7	86.7
Yes	305	245	80.3	83
**Sarcopenia + obesity**				
No	590	465	85	75.4
Yes	31	25	75.4	85.7
**Diabetes mellitus**				
No	508	402	79.5	86.6
Yes	114	88	77.2	75.8
**Decompensatio cordis**				
No	591	466	78.9	84.8
Yes	30	24	80	78.7
**COPD**				
No	591	454	78.41	84.9
Yes	42	36	80	78.2
**Charlson comorbidity index**				
0	336	266	79.2	88.8
1+	283	223	78.9	78.8
**ASA score**				
I + II	526	409	78.1	83.7
III + IV	92	77	83.7	87.5
**CEA**				
< 7ug/L	430	360	83.7	87.8
≥ 7ug/L	146	93	63.7	73.1
**Tumor location**				
Colon	444	371	83.6	85.8
Rectum	177	119	67.2	80
**T-stage**				
1	36	34	94.4	92.2
2	193	171	88.6	92.1
3	362	270	74.6	81.2
4	30	15	50	62.3
**N-stage**				
0	336	303	90.2	92.7
1	134	103	76.9	82.9
2	80	33	41.3	50.1
X	71	51	71.8	83.4
**Tumor stage**				
I	180	164	91.1	94.6
II	227	190	83.7	88.4
III	214	136	63.6	71
**Tumor grade**				
Good	79	66	83.5	86.5
Moderate	470	377	80.2	85.7
Poor	53	33	62.3	73.1
Unknown/other	10	7	70	79.7
**Radicality**				
R0	605	477	80	84.5
R1	15	12	78.8	77.5
**Postoperative complications**				
No	266	298	83.9	90.4
Yes	355	192	72.2	76.8
**Readmission < 30 days**				
No	552	438	79.5	94.1
Yes	68	52	76.5	87.1
**Neoadjuvant therapy**				
No	462	383	82.9	85.7
Yes	158	106	67.1	79.9
**Adjuvant chemotherapy**				
No	127	382	78.1	84
Yes	489	104	81.9	86.1

The observed cure rate in patients aged ≥ 70 years was 74.7%, versus 82.3% for patients aged < 70 years. The observed cure rate for women was slightly higher than that for men (81.8% versus 76.4%). The observed cure rate for patients with a T4-tumor was notably low at 50% and considerably higher for patients with a T1-tumor (94.4%), T2-tumor (88.6%), and even those with a T3-tumor (75.6%). Patients with a N2-tumor had the lowest observed cure rate, viz. only 41.3%, as opposed to 90.2% for patients with N0-tumor and 76.9% for patients with an N1-tumor. The observed cure rate for patients with stage III CRC was 63.6%, almost 30% lower than that for patients with stage I disease (91.1%). Furthermore, the observed cure rate for patients with a poor tumor grade was only 62%—in line with that for patients with CEA ≥ 7 μg/L (64%) and patients with rectal cancer (67%).

The predicted cure rate of 80.1% for patients ≥ 70 years of age is slightly higher than the observed cure rate for this group (Table 2). Patients with a T1- and/or N0-tumor had the highest probability of cure, i.e., 94.4% and 90.2%, respectively. Conversely, patients with a T4-tumor or N2-tumor had the lowest probability of cure, i.e., 62.3% and 50.1%, respectively. The predicted probability of cure for tumor stage was 94% for stage I, 88% for stage II, and 71% for stage III. Regarding type of cancer, colon cancer was associated with a higher probability of cure than is rectal cancer (85.5% versus 80%). Two other factors were associated with a relatively low probability of cure, i.e., preoperative CEA level of > 7uq/L and poor tumor differentiation (both 73%). The predicted cure rate of patients who experienced postoperative complications was 76.8%. Although underweight BMI and diabetes were not significantly associated with CSS in univariate analyses, both had a relatively low probability of cure in the mixture cure model (75.6% and 73.1%, respectively). Nonetheless, patients who did not have postoperative complications and had not been readmitted within < 30 days postoperatively had a probability of cure of over 90% (90.4% and 94.1%, respectively).

## Discussion

The findings of this multicenter cohort study are consistent with failure of the curative intent treatment strategies for stage I–III CRC in 35% of cases. Age > 70 years at diagnosis, high preoperative CEA level, rectal cancer, high T-and N-stage, high tumor stage, poor tumor differentiation, and postoperative complications were all individual poor prognostic factors for cancer-specific survival after surgery for stage I–III CRC. Observations and mixture cure model analysis showed that patients with T4-stage, N2-stage, stage III CRC, CEA level ≥ 7 μg/L, and poor tumor differentiation had the lowest chance of eventual cure. Nevertheless, the clear majority of 5-year survivors (65%) had no evidence of disease or had died of a non-cancer related cause and could therefore be defined as cured. Patients with a T1-stage tumor, N0-stage tumor, tumor stage I, and/or postoperative complications had the highest probability of cure (> 90%).

In the Netherlands and most other countries, once a patient has remained free from recurrence of disease for 5 years after surgery, the medical community considers many cancers “cured” [7]. Although recurrence of disease after 5 years is not impossible, the probability of this happening is very low. Therefore, follow-up programs are usually limited to 5 year postoperatively [10, 12].

We found that the pathologic tumor characteristics were the most important indicators of probability of cure. The probability of cure for patients with a T1-tumor was 92%, which decreased to 62% for patients with a T4-tumor. Correspondingly, the probability of cure for patients with an N0-tumor was 93%, which decreased markedly to 50% for patients with an N2-tumor. These findings are in line with previous research by Gunderson and colleagues, who showed a 5-year survival rate of 97% for both patients with a T1N0 tumor and patients with T2N0 tumor, compared with 55% for patients with a T4N0 tumor and 56.8% for patients with a T1N2 tumor [23]. Tumor grade is generally considered a stage-independent prognostic factor for survival, in that poor differentiated tumors are associated with poor patient survival [24, 25]. In the current study, poorly differentiated tumors were associated with a significantly lower survival rate (62%) and predicted cure (73%) than were well-differentiated tumors (83% and 87% respectively). These results highlight the importance of especially T- and N-staging in non-metastatic CRC, seeing that current treatment strategies that have a curative intent are insufficient for a subgroup of patients with poor tumor characteristics. Some studies have found encouraging survival outcomes in patients with a T4-tumor with the use of proactive strategies, such as the second-look approach [26, 27] and prophylactic resection of target organs for peritoneal metastases during the first surgery [28]. However, two large phase III trials failed to show benefit from adjuvant intraperitoneal hyperthermic chemoperfusion (HIPEC) in high-risk patients [29, 30]. An effective treatment for high-risk patients is therefore still needed.

The present study findings complement earlier results in in that they associate older age with poorer cancer-specific survival [31, 32]. Provision of less intensive therapy to the elderly or the elderly refusing treatment may have resulted in higher recurrence rates and causal death [33–36].

In line with previous literature, patients with rectal cancer had significantly lower chances of long-term survival and cure than had patients with colon cancer [37]. Sex, for which literature shows contradicting results, was not a prognostic factor for survival [38–42]. Furthermore, we found no association between the presence of comorbidities and CSS. Diabetes, congestive heart failure, and COPD, but also grading systems for comorbidities such as the ASA score and the CCI index, were not associated with CSS in our study. The literature on the association between comorbidities and CSS is somewhat contradictory. While some studies found a lower survival with increasing comorbidity, other studies found the association differs between colon cancer and rectal cancer [43, 44]. The reasons underlying these results in other studies have not been elucidated, although possible contributors include under-treatment and reduced resilience to cope with cancer effects and treatment toxicity.

This is, to our knowledge, the first study to provide unique estimates of the likelihood of both observed and predicted cure depending on particular risk factors in a large Dutch prospective multicenter study on stage I–III colorectal cancer patients.

However, this study has several limitations. In general, surveillance imaging had been performed at least every 6 months after surgery. The time interval of 6 months may have led to lead time bias. As mentioned in the results, for 85 patients with an N0 tumor, less than 10 lymph nodes were dissected or pathologically analyzed which could have led to wrong nodal staging. This could inherently lead to survival differences if some of these patients did actually have lymph node metastases. Furthermore, we did not address molecular tumor characteristics that are related to survival, which play an increasingly bigger role in the prediction of survival in CRC [45].

In conclusion, while CRC is recognized as a possible fatal malignancy, a substantial improvement on therapies and thereby survival of patients with CRC has been accomplished over the recent years. Appropriate survival analysis like the mixture cure rate model performed in this study can help the clinicians and researchers in identifying potential risk factors, which affect the survival and cure fraction of patients who are not susceptible to death from CRC. This mixture cure model provides a framework to compare both patient-related and treatment-related prognostic factors and to gives valuable insight in the probability of being cured of CRC for each of these variables. The probability of cure for patients with stage I–III colorectal cancer included in this study ranges from 50 to 94%. Even with poor prognostic factors, such as high tumor stage and poor differentiation grade, cure is highly likely with standard therapy consisting of surgery and adjuvant or neoadjuvant systemic therapy when indicated. Still, this is less obvious for older patients with high T- and N-stage tumors and/or poor tumor differentiation. Instead of only providing patients with overall 5-year survival rates, with general patient characteristics, this cure model can aid physicians in providing a more individualized prognosis and chance of curation from this disease.

## Data Availability

The data that support the findings of this study are available on request from the corresponding author. The data are not publicly available due to privacy or ethical restrictions.
