# Correction to: Actual survival after resection of primary colorectal cancer: results from a prospective multicenter study

**DOI:** 10.1186/s12957-021-02252-z

**Published:** 2021-05-26

**Authors:** Inge van den Berg, Robert R. J. Coebergh van den Braak, Jeroen L. A. van Vugt, Jan N. M. Ijzermans, Stefan Buettner

**Affiliations:** Department of Surgery, Erasmus MC - University Medical Center Rotterdam, Rotterdam, 3015 GD The Netherlands

**Correction to: World J Surg Onc 19, 96 (2021)**

**https://doi.org/10.1186/s12957-021-02207-4**

Following publication of the original article [1], the authors reported that there was an error in reference 30.

Currently it reads:

Charlotte KDD, Cornelis J, Petur S, Johannes C, Arend A, Bemelman WA, et al. Adjuvant HIPEC in patients with colon cancer at high risk of peritoneal metastases: Primary outcome of the COLOPEC multicenter randomized trial. J Clin Oncol. 2019;37:482.

It should read:

Klaver CEL, Wisselink DD, Punt CJA, Snaebjornsson P, Crezee J, Aalbers A, et al. Adjuvant HIPEC in patients with colon cancer at high risk of peritoneal metastases: Primary outcome of the COLOPEC multicenter randomized trial. J Clin Oncol. 2019;37:482.

Additionally, a minor change in the abstract has been made and Figure 2I and 2J has been updated with the better resolution figure.

The Original article has been updated.
